# Knowledge and practices of Iranian students (13–16 age) regarding the use of fluoride toothpaste

**DOI:** 10.1186/s12903-024-04513-1

**Published:** 2024-06-28

**Authors:** Maryam Alsadat Hashemipour, Zahra Zeyghami, Halle Rajaee

**Affiliations:** Social Determinants on Oral Health Research Center, Kerman University of Medical Sciences, Kerman, Iran

**Keywords:** Fluoride, Knowledge, Performance, Student, Toothpaste

## Abstract

**Introduction:**

Fluoride is the main factor in reducing the prevalence of caries worldwide. However, there is insufficient knowledge about whether people in different age groups are aware of the benefits of fluoride toothpaste, as well as about people’s daily oral care habits and whether they use fluoride. The purpose of this research is to investigate the knowledge and performance of Iranian students regarding the use of toothpaste containing fluoride.

**Method:**

This study was conducted on the first- and second-year high school students of Kerman city. Questionnaires containing personal questions, general questions, and questions related to students’ knowledge and performance regarding the use of fluoride toothpaste were provided to them and then they were asked to complete and submit it. The results obtained from the survey were analyzed by T-test, Mann-Whitney test, and Chi-Square test in SPSS Version 24. The significance level in data analysis was *P* < 0.05.

**Results:**

In this research, 681 forms including 252 boys and 429 girls were examined. The average age of the participants was 14.1 ± 0.4. 91.2% declared that they use toothpaste and 77.8% of them used toothpaste containing fluoride. 521 people stated that the price of toothpaste is important in using the type of toothpaste. 621 people used regular toothbrush and 609 people evaluated their oral health as good. 621 of the participants stated that toothpaste makes the mouth healthy. The average knowledge score was 16.7 ± 2.1 out of 24, which indicates the average knowledge of students in this field.

**Conclusion:**

This study showed that students’ knowledge and performance about using fluoride-containing toothpaste is average. There was no significant relationship between performance and knowledge with age and gender. There was also a positive correlation between knowledge and performance indicating that increasing knowledge leads to an increase in behavioral changes. Also, there was a positive correlation between knowledge and performance, and a correlation coefficient of 0.731 was obtained between knowledge and performance. It shows that increasing knowledge leads to increasing behavioral changes.

## Introduction

Dental caries is still a major public health problem that affects people of all ages in most countries. When fluoride toothpaste was introduced in the 1960s, caries only showed a decreasing trend in Sweden, while in the rest of the Western world, it took several decades to see a reduction in caries incidence. There are indications that caries, especially among growing individuals and the elderly population, is on the rise again [1].

Fluoride toothpaste is one of the main reasons for the decrease in the prevalence of dental caries worldwide [2]. However, there is not enough knowledge about whether individuals in different age groups are aware of the benefits of fluoride toothpaste or not, as well as their daily oral care habits and whether they use fluoride or not [3, 4].

Fluoride toothpaste is widely advertised globally for cosmetic purposes. This trend is in line with the legal definition of regular fluoride toothpaste as a cosmetic and hygiene product, in fact, teenagers use toothpaste for cosmetic purposes. On the other hand, dentists promote fluoride toothpaste due to its therapeutic effect in preventing dental caries. An increasing number of children and teenagers request teeth whitening [5].

Brushing teeth with fluoride toothpaste is considered equally important as fluoride for preventing dental caries. Dental professionals used to think that identifying plaque on teeth is easier for their patients than asking about their use of fluoride toothpaste. The reason for this was that they assumed their patients already had the necessary knowledge about fluoride toothpaste and its use. However, individual intervention by a dental health professional has a significant impact on the use of fluoride toothpaste [6]. One of the most effective ways to prescribe fluoride is through regular use of fluoride toothpaste [7–9]. Therefore, the aim of this study was to investigate the knowledge and performance of Iranian students regarding the use of fluoride toothpaste.

## Method

This research is a cross-sectional analytical study (Clinical Trial Number: 401,000,590). The target population in this study was the first- and second-year high school students in Kerman city. First, the names of schools were obtained from the Department of Education and then, using cluster sampling, four schools (two girls’ schools and two boys’ schools) from each education district were selected. Then two classes were selected from each school and questionnaires were distributed (z: 1.96; p = q = 0.3; d: 0.03, Number of samples: 700).

The researcher-made questionnaire (Persian language) contained personal questions such as age, gender, and year of enrollment, general questions, and questions related to students’ knowledge and performances about the use of fluoride toothpaste, which were given to students to complete and submit. The nature and purpose of this study were such that students who entered the study, were allowed to leave the study at any time after the interview began. The purpose of this research was explained to each individual, and if they wished, the questionnaire was provided to them. In addition, all individuals were assured that the information on the questionnaire would remain confidential and would only be used for statistical purposes.

The questions in this questionnaire were designed by two dental professionals (Oral Medicine and Operative Dentistry) and one statistics expert.

The scientific validity of the questionnaire was confirmed by providing it to six dental professionals experts, and the level of question content and comprehensibility was discussed. Based on their opinions and text analysis, the content validity of the questionnaire was satisfactory. However, two knowledge questions and two performance questions were removed according to their opinion.

Then, to evaluate the reliability, first, the questionnaire was given to ten students, and then two weeks later, the questionnaire was given to them again. The reliability of the questionnaire was satisfactory regarding the Cronbach’s alpha coefficient of 0.76.

In terms of knowledge questions, each correct answer received 2 points, each wrong answer received 0 points, and “I do not know” received 1 point. The knowledge questions included 12 questions, so the knowledge score ranged from 0 to 24 (Good = 20–24, Average = 10–19, Poor = 0–9). In terms of performance questions, each correct answer received 2 points, each wrong answer received 0 points. The performance questions included 14 questions, so the performance score ranged from zero to 28 (Good = 23–28, Average = 13–22, Poor = 0–12).

## Results

The results obtained from the analysis by T-test, Pearson correlation coefficient, Chi-square test, and SPSS version 24 were analyzed. The level of significance in data analysis was *P* < 0.05.

In this study, 700 forms were completed by students, and 681 forms were analyzed (Response Rate = 97.3%). Of these, 252 (37%) were boys and 429 (63%) were girls. The average age of participants was 14.1 ± 0.4. The minimum age was 13 and the maximum age was 16. Table 1 621 participants (91.2%) reported using toothpaste, and 453 participants (77.8%) reported using fluoride toothpaste.

**Table 1:** Demographic characteristics of participating in the study

Characteristics		Male		Female		Total	
		No	%	No	%	No	%
Sex		252	37	429	63	681	100
Age	Min	13		13		13	
	Max	16		14		16	
Mean age		14.4 ± 0.3		13.8 ± 0.1		14.1 ± 0.4	
Academic year (high school)	First	125	18.3	237	34.9	362	53
	Second	127	18.7	192	28	319	46.9

Table 2 shows the participants’ responses to general questions. 521 participants (76.51%) reported that the price of toothpaste is important in choosing a type of toothpaste. 621 participants (91.19%) used a regular toothbrush. 312 participants (45.81%) reported receiving education on using toothpaste, and in 212 cases (3028%), this education was provided by parents. 609 participants (87%) evaluated their oral health as good.

**Table 2:** Answers of participants to general questions

Questions		No	%
What determines which toothpaste to use?	Price	521	76.51
	Taste	402	59.03
	Advertising	102	14.98
Do you use a regular toothbrush or an electric toothbrush?	Normal toothbrush	621	91.19
	Electric toothbrush	58	8.52
	Both	2	0.29
Has anyone ever shown or instructed you how to use toothpaste?	Yes	312	45.81
	No	369	54.19
How do you evaluate your oral health?	Good	609	89.4
	Bad	52	7.64
	Medium	20	2.9
What is important to you about your teeth?	Healthy teeth	239	35.10
	White teeth	317	46.55
	No caries	125	18.36
Do you think brushing with toothpaste is important?	It’s too important	337	49.49
	It is important	298	43.76
	No matter	34	4.99
	I do not know	12	1.76
What is the importance of fluoride in toothpaste?	Teeth whitening	324	47.58
	Strengthens teeth	402	59.03
	Makes the teeth cleaner	346	50.81
	It gives the mouth a fresh feeling	123	18.06
Which one do you think is important against tooth decay?	Cleaning with a toothbrush	284	41.70
	Fluoride in toothpaste	246	36.12
	Toothbrush and toothpaste are equally important	151	22.17

Table 3 shows the participants’ responses to knowledge questions. 621 participants (91.19%) stated that toothpaste promotes oral health. 567 participants (81%) stated that if someone uses toothpaste, it will have less effect on their teeth’s sugar level. 501 participants (71.57%) correctly stated that using toothpaste can make teeth less yellow. The mean ± SD of knowledge score was 16.7 ± 1.2 out of 24, indicating the average level of knowledge among students in this field.

**Table 3:** Answers of participants to knowledge questions

Questions	Correct		No correct		No idea	
	No	%	No	%	No	%
Toothpaste makes the mouth healthy.	621	91.2	39	5.7	21	3
Toothpaste makes teeth stronger.	602	88.3	68	10	11	1.6
Toothpaste kills bacteria.	621	91.2	60	8.8	0	0
Toothpaste prevents tooth decay.	611	89.7	62	9.1	8	1.2
If one uses toothpaste, the sugar will have less effect on the teeth.	567	83.3	42	6.2	72	7.6
Toothpaste protects against acid because toothpaste is alkaline and increases pH.	512	75.2	72	10.5	97	14.2
Fluoride is the substance in toothpaste that prevents gum disease.	511	75	71	10.4	36	5.3
Fluoride destroys the bacteria inside the teeth.	541	79.5	55 8	8	22	3.2
Using toothpaste is desirable to prevent dental diseases and tooth loss.	591	86.8	59	5.9	31	4.5
In order for brushing to become a habit, one must brush for a long time.	569	83.5	97	14.3	15	2.2
Using toothpaste can make your teeth less yellow.	501	73.5	155	22.3	25	3.6
Dirty and ugly teeth are a sign of poor personal hygiene.	642	94.3	36	5.3	3 4	0

**Fig. 1:**
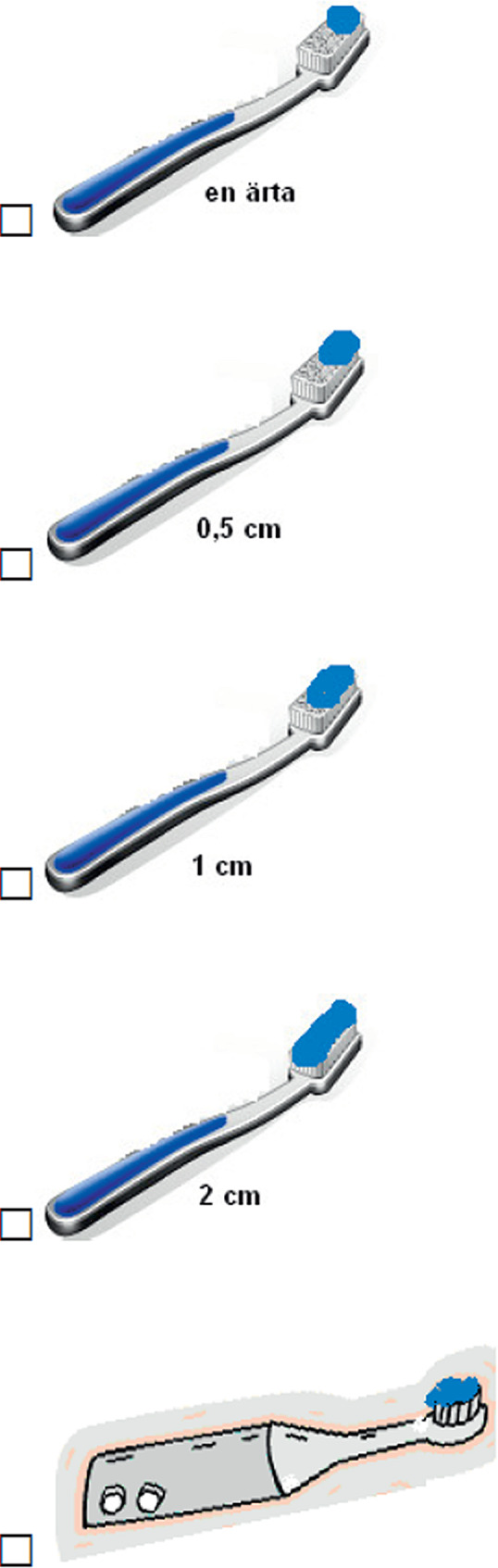
How much toothpaste do you use when you brush your teeth?

Figure 1 show the amount of toothpaste used by students. This study showed that 402 participants (57.42%) used half a centimeter of toothpaste. 102 participants (14.57%) used 2 centimeters of toothpaste, and 112 participants (16%) used 1 centimeter of toothpaste.

Table 4 shows the participants’ responses to performance questions. 543 participants (77.57%) stated that they brush their teeth at least once a day, and 101 participants (14.43%) brushed their teeth twice a day. 502 participants (71.71%) reported brushing their teeth for less than a minute. In this study, only 60 participants (8.9%) used mouthwash after brushing their teeth. 102 participants (14.57%) stated that they clean between their teeth using dental floss.

**Table 4:** Participant responses to performance questions

Questions		No	%
How many times a day do you brush your teeth?	Never	25	3.6
	1 time	543	79.7
	2 times	101	14.8
	3 times or more	12	1.7
When do you brush your teeth?	Before breakfast	145	21.3
	After breakfast	123	18
	Before dinner	5	0.76
	after dinner	62	9.1
	Before I go to sleep	341	50
	In the evenings	5	0.73
Does it happen that you don’t brush your teeth in the morning or at night?	Yes	202	29.6
	No	479	70.33
Do you drink or eat anything after brushing your teeth?	Yes	502	73.7
	No	179	26.3
Do you use anything else after brushing your teeth, such as lozenges or medicines?	Yes	179	26.3
	No	502	73.7
Do you always brush your teeth with toothpaste?	Yes, in general	188	27.6
	No, not every time	404	59.3
	No never	89	13
Do you use fluoride or non-fluoride toothpaste?	With fluoride	453	66.5
	Without fluoride	216	31.7
	I do not know	12	1.76
How often do you not brush your teeth with toothpaste?	Once a day	252	37
	More than 2 times a week	189	27.7
	Once a week	145	21.3
	Sometimes in the month	89	13
	Never	6	0.88
How do you clean between your teeth?	Toothbrush	45	6.6
	Dental floss	102	14.5
	Toothpick	12	1.76
How long do you brush your teeth?	Less than 1 min	502	73.3
	1 min or more	179	26.3
How many times do you dip your toothbrush under running water when you brush your teeth?	2 times and less	567	83.2
	More than 2 times	114	15.4
Do you rinse your mouth with water after brushing your teeth?	No never	82	12
	Yes, every once in a while	56	8.22
	Yes, more	34	4.5
	Yes, always	509	74.7
If you rinse your mouth after brushing your teeth, how much water do you use?	One fist	309	54.6
	Two fists	160	23.5
	Half a glass of water	156	22.9
	A glass full of water	56	8.22
Do you rinse your mouth with a solution (mouthwash) afterwards?	Yes	60	8.8
	No	621	0.91

The average performance score was 18.10 ± 2.22 out of 28, indicating a relatively average performance level among study participants. This study showed that there was no significant relationship between performance score and age or gender. Table 5 there was also a positive correlation between knowledge and performance, with a correlation coefficient of 0.731, indicating that increasing knowledge leads to increased behavioral changes.

**Table 5:** The relationship between knowledge, performance and demographic charecteristics in participants

Variable		Mean Knowledge	*P* Value	Mean Performance	*P* Value
Sex	Male	14.1 ± 1.1	0.01*	18.12 ± 2.12	0.09
	Female	18.04 ± 1.2		18.08 ± 2.32	
Age	< 14	14.2 ± 0.2	0.01*	18.01 ± 2.02	0.31
	≥ 14	17.9 ± 2.2		18.19 ± 2.42	
Use a regular toothbrush	Yes	16.2 ± 1.1	0.12	21.10 ± 2.32	*0.01
	No	16.1 ± 0.9		15.10 ± 2.10	
Oral health	Good	17.5 ± 1.1	0.001*	20.15 ± 2.31	0.01*
	Not good	15.09 ± 0.2		16.10 ± 2.11	
Brushing with toothpaste	Yes	16.1 ± 1	0.21	21.12 ± 2.31	0.02*
	No	16.2 ± 1.2		15.08 ± 2.11	
Academic year (high school)	First	16.03 ± 1.1	0.06	18.11 ± 2.15	0.05
	Second	16.11 ± 1.3		18.09 ± 2.30	

*P < 0.05 is significant

## Discussion

In this study, 543 participants (79.7%) reported brushing their teeth at least once a day, and 101 participants (14.8%) brushed their teeth twice a day.

In the field of oral hygiene, one of the constant concerns of researchers in this field is daily brushing, and it should be done correctly by every person at least twice a day. According to the finding by Zhu et al., preventive measures are more effective than curative measures [10]. While it is beneficial that more than half of students brush their teeth twice a day, there is a need to increase knowledge about oral hygiene practices, including the recommended amount of toothpaste and frequency of brushing.

A meta-analysis study showed that brushing teeth more than twice a day or less than twice a day does not have a significant effect on the occurrence of dental caries, but they noted that dental caries occur more frequently in primary teeth compared to permanent teeth [6]. Another study showed that the strongest evidence related to dental caries in 12-year-olds is found in the frequency of brushing and dental plaque [11].

In this research, 502 participants (73.7%) reported brushing their teeth for less than a minute. Also, 621 participants (91.2%) reported using toothpaste, and 453 participants (77.8%) reported using fluoride toothpaste.

The Saveanu et al. study [12] showed that about half of the participants did not know the recommended amount of toothpaste to use while brushing. Only a quarter of individuals considered fluoride content when choosing toothpaste. About one-third of students did not know proper brushing techniques and chose their toothbrush based on criteria other than dental guidelines.

The mean ± SD of knowledge score was 16.7 ± 1.2 out of 24, indicating the average level of knowledge regarding the use of fluoride toothpaste among students.

The Saveanu et al. study [12] showed that most students have at least basic knowledge about the frequency, purpose, and timing of brushing. Only about 20.47% of students knew about the beneficial effects of fluoride in toothpaste on mineralizing dental tissue. This lack of knowledge about fluoride and its benefits among students has been shown in other studies as well [13–18]. A study showed that Australian preschoolers who used non-fluoride toothpaste had a higher rate of dental caries compared to other regions worldwide [19]. A Cochrane review supports the benefits of using fluoride toothpaste in preventing dental caries compared to non-fluoride toothpaste [20].

The fact that a significant percentage of students do not have knowledge about the amount of toothpaste to use and the frequency of brushing has been shown in other specialized studies as well [19–22].

The benefits of fluoride-containing toothpaste are well established. Overall, studies are relatively high quality and provide clear evidence that fluoride-containing toothpaste is effective in preventing dental caries [9]. It appears that fluoride-containing toothpaste, frequency of brushing, fluoride concentration in toothpaste, amount of toothpaste on the brush, brushing time, and rinsing with water are effective factors in this area [23–26].

Differences in studies can be caused by the studied population, the studied country, the level of oral health of the community and the culture of the community.

Although fluoride-containing toothpaste is generally available in northern European countries, around 25% of 14-year-old adolescents do not brush their teeth daily [27, 28]. In Sweden, 90% of adult report brushing their teeth at least once a day [29]. The recent studies showed that almost half of adults brush their teeth for more than two minutes [30, 31] where brushing time was observed to be slightly over one minute. In the study by Koivusilta et al. [28], only 9% of adults used proper rinsing technique after brushing.

This study showed that in 402 participants (56%), the amount of toothpaste used was half a centimeter. 102 participants (15%) used 2 centimeters of toothpaste, and 112 participants (14.5%) used 1 centimeter.

The American Academy of Pediatrics, the American Academy of Pediatric Dentistry, and the American Dental Association recommend fluoride-containing toothpaste for all children and limit the amount of toothpaste used by children under 3 years old to a “smear” (a grain of rice) [12–14].

According to Hu et al. controlling the amount of toothpaste consumption is important in reducing the risk of fluorosis. A meta-analysis showed that using a pea-sized amount minimizes the risk of fluorosis in children and maximizes the preventive benefits of tooth decay for all age groups [22].

In this study, 621 participants (91.2%) stated that toothpaste promotes oral health. 567 participants (83.2%) claimed that if someone uses toothpaste, it will have less effect on their teeth. The use of toothpaste can whiten teeth less by 501 participants (73.6%).

Furundzic et al. [7] showed that reasons for using fluoride-containing toothpaste in students include oral health, a substance that strengthens teeth and eliminates bacteria and therefore prevents decay. Additionally, if someone uses toothpaste, it will have less effect on their teeth, and toothpaste can be used to protect against acid because toothpaste is alkaline and therefore increases PH which is compatible with other studies [25, 26, 28–30].

Differences in studies can be caused by the studied population, the studied country, the level of oral health of the community and the culture of the community.

This study showed that the average knowledge and performance scores of students were moderate, and there was no significant correlation between performance and knowledge scores with age and gender.

There are multiple studies in specialized articles that analyze the relationship between students’ knowledge level and their knowledge and performance regarding oral hygiene [3, 4]. Kamran et al. [25] showed that women have better grades in knowledge, attitude, and behavior toward oral hygiene compared to men, which is consistent with other studies [27–30].

There was also a positive correlation between knowledge and performance, and a correlation coefficient of 0.731 was obtained between knowledge and behavior, which indicates that increasing knowledge leads to an increase in behavioral changes that is consistent with the research of Kamran and colleagues [25].

Eleven studies showed a significant positive effect of intervention on awareness, attitude, and behavior (brushing and flossing) of the experimental group. Nakre and Harikiran suggested that involving other groups such as parents and teachers in oral health education and promotion is more effective. Increasing knowledge can improve oral health education [21].

## Conclusion

This study showed that students’ knowledge and performance about using fluoride-containing toothpaste is average. There was no significant relationship between performance and knowledge with age and gender. There was also a positive correlation between knowledge and performance that indicating that increasing knowledge leads to an increase in behavioral changes.

## Limitation

Non-cooperation of the number of participants.

Non-cooperation of a number of school officials.

Incomplete filling of a number of questionnaires.

## Data Availability

No datasets were generated or analysed during the current study.

## References

[1] Bagramian RA, Garcia-Godoy F, Volpe AR (2009). The global increase in dental caries. A pending public health crisis. Am J Dent.

[2] World Health Organization. WHO/FDI/IADR Global consultation on use of fluoride for oral health,2006.

[3] Marinho VCC, Higgins JPT, Sheiham A, Logan S (2009). One topical fluoride (toothpaste, or moutrinses, or gels, or varnishes) versus another for preventing dental caries in children and adolescents. Cochrane Database Syst Rev.

[4] Bunkiene V, Aleksejüniene J (2009). An overview of oral health promotion in adolescents. Int J Paediatr Dent.

[5] Featherstone JD (2004). The continuum of dental caries – evidence for a dynamic disease process. J Dent Res.

[6] Petersen PE, Baez R, Kwan S, Ogawa H. Future Use of Materials for Dental Restoration: Report of the Meeting convened at WHO HQ, Geneva, Switzerlannd,16th to 17th November 2009. World Health Organization;2009.P.1.

[7] Furundzic K, Malmberg J, Sandström B, Ericson D (2020). Why do adolescents use Fluoride Toothpaste? A qualitative interview investigation. Oral Health Prev Dent.

[8] Petersen PE, Bourgeois D, Ogawa H, Estupinan-Day S, Ndiaye C (2005). The global burden of oral disease and risks to oral health. Bull World Health Organ.

[9] Marinho VC, Higgins JP, Sheiham A, Logans S. Fluoride toothpastes for preventing dental caries in children and adolescents. Cochrane Database Syst Rev 2003;CD002278. 10.1002/14651858.CD002278 PMC8439270 12535435

[10] Den Beste P, Ko HS (1996). Fluoride levels in whole saliva of pre-school children after brushing with 0,25 g (pea-sized) as compared to 1,0 g (full-brush) of fluoride toothpaste. Pediatr Dent.

[11] Davies RM, Ellwood RP, Davies GM (2003). The rational use of fluoride toothpaste. Int J Dent Hyg.

[12] Sjögren K, Birkhed D (1994). Effect of various post-brushing activities on salivary fluoride concentration after toothbrushing with sodium fluoride dentifrice. Caries Res.

[13] Saveanu CI, Cretu CC, Bamboi I, Săveanu AE, Anistoroaei D (2022). Title cross-sectional study to evaluate knowledge and attitudes on oral Hygiene of Romanian Students. Medicina.

[14] Tichenor M, Sridhar D. Metric partnerships: global burden of disease estimates within the World Bank, the World Health Organisation and the Institute for Health Metrics and evaluation. Wellcome Open Res. 2019;4. 10.12688/wellcomeopenres.15011.2 PMC6406176 30863794

[15] Peres MA, Macpherson L, Weyant RJ, Daly B, Venturelli R, Mathur MR (2019). Oral diseases: a global public health challenge. Lancet.

[16] Hosseinpoor AR, Itani L, Petersen PE (2012). Socio-economic inequality in oral healthcare coverage: results from the World Health Survey. J Dent Res.

[17] Ending Childhood Dental Caries. WHO implementation Manual. World Health Organization: Geneva, Switzerland,; 2019.

[18] Al-Qahtani SM, Razak PA, Khan SD (2020). Knowledge and Practice of Preventive Measures for Oral Health Care among male Intermediate Schoolchildren in Abha, Saudi Arabia. Int J Environ Res Public Health.

[19] Hou R, Mi Y, Xu Q, Wu F, Ma Y, Xue P (2014). Oral health survey and oral health questionnaire for high school students in Tibet, China. Head Face Med.

[20] Wahengbam PP, Kshetrimayum N, Wahengbam BS, Nandkeoliar T, Lyngdoh D (2016). Assessment of oral health knowledge, attitude and self-care practice among adolescents—A state wide cross- sectional study in Manipur, North Eastern India. J Clin Diagn Res.

[21] Buckeridge A, King N, Anthonappa R (2021). Relationships between parental education, choice of child dentifrice, and their children’s caries experience. Int J Ped Dent.

[22] Walsh T, Worthington HV, Glenny AM, Marinho VC, Jeroncic A. Fluoride toothpastes of different concentrations for preventing dental caries. Cochrane Database Syst Rev. 2019;3: CD007868. 10.1002/14651858.CD007868.pub3 PMC6398117 30829399

[23] Creeth J, Bosma ML, Govier K (2013). How much is a ‘pea-sized amount’? A study of dentifrice dosing by parents in three countries. Int Dent J.

[24] Nordström A, Birkhed D (2017). Attitudes and behavioural factors relating to toothbrushing and the use of fluoride toothpaste among caries-active Swedish adolescents-A questionnaire study. Acta Odontol Scand.

[25] Kamran A, Bakhteyar K, Heydari H, Lotfi A, Heydari Z (2014). Survey of oral hygiene behaviors, knowledge and attitude among school children: a cross-sectional study from Iran. Int J Health Sci.

[26] Zero DT, Creeth JE, Bosma ML, Butler A, Guibert RG, Karwal R (2010). The effect of brushing time and dentifrice quantity on fluoride delivery in vivo and enamel surface microhardness in situ. Caries Res.

[27] Zhu LPP, Wang HY, Bian JY, Zhang BX (2005). Oral health knowledge, attitudes and behaviour of adults in China. Int Dent J.

[28] Peterson P, Esheng Z (1998). Dental caries and oral health behavior situation in children and school children in Wuham. People Repub China Int Dent J.

[29] Singh A (2009). Oral health knowledge, attitude and practice among NCC Navy Cadets and their correlation with oral hygiene in south India. Oral Health Prev Dent.

[30] Prashanth ST, Bhatnagar S, Das UM, Gopu H (2011). Oral health knowledge, practice, oral hygiene status, and dental caries prevalence among visually impaired children in Bangalore. J Indian Soc Pedo Prev Dent.

[31] Khami MR, Virtanen JI, Jafarian M, Murtomaa H (2007). Oral health behaviour and its determinants amongst Iranian dental students. Eur J Dent Educ.

